# Phylogenetic analysis of swine influenza A (H1N2) viruses isolated in Jinju City, Republic of Korea

**DOI:** 10.1128/MRA.00549-23

**Published:** 2023-09-25

**Authors:** Min-Ji Kim, MiJung Kwon, Min Ji Kim, Eui Hyeon Lim, Bang-hun Hyun, Yoon-Hee Lee, Seong-In Lim

**Affiliations:** 1 Viral Disease Division, Animal and Plant Quarantine Agency, Gimcheon, Gyeongsangbuk-do, South Korea; Queens College Department of Biology, Queens, New York, USA

**Keywords:** swine influenza, phylogenic analysis, H1N2

## Abstract

Genomic sequences of the swine influenza A (H1N2) viruses “A/Swine/South Korea/GN-1/2018” and “A/Swine/South Korea/GNJJ/2020” sampled from Jinju City, Republic of Korea, are reported here. The sequences of these viruses were 99% similar. These included eight genes from each of the H3N2pM, A(H1N1)2009pdm, and North American swine lineages.

## ANNOUNCEMENT

The swine influenza virus (SIV) belongs to the genus *Alphainfluenza* in the family *Orthomyxoviridae*. SIV consists of polymerase bases 1 and 2 (PB1 and PB2), polymerase acid (PA), hemagglutinin (HA), nucleoprotein (NP), neuraminidase (NA), matrix (M), and nonstructural protein (NS) (1, 2). Currently, three viral subtypes (H1N1, H1N2, and H3N2) circulate in pigs (3). Viruses continuously change and generate new viral subtypes yearly via mutation, recombination, and reassortment (4). Therefore, it is necessary to monitor influenza A virus genome diversity through ongoing epidemiological studies to respond to it.

This article presents the complete coding genomes of two H1N2 influenza viruses obtained in 2018 and 2020 from nasal swabs taken from a slaughterhouse in Jinju City, Republic of Korea. Viral RNA was extracted using the RNeasy Mini Kit (Qiagen, Hilden, Germany). The strains were identified as SIV through RT-PCR amplification of the common M gene using the VDX SWIAV NFMP RT-PCR Kit (Median Diagnostics, Kangwon-do, Republic of Korea) (5). To isolate the virus, positive nasal swabs were submerged in pH 7.4 phosphate-buffered saline (Thermo Fisher Scientific, Waltham, MA, USA); this suspension was then filtered using a 0.45 µm syringe filter (Pall Corporation, Port Washington, NY, USA) and inoculated in specific-pathogen-free eggs (Valo, Boston, MA, USA) (6, 7). The allantoic fluid was collected after 3 days and verified using a hemagglutination assay. Eight viral genes encoding the main components of SIV, as previously mentioned, were amplified via One-Step RT-PCR (Qiagen) using reference primers (6, 8, 9) and sequenced by the Sanger technique using ABI 3730xl devices at commercial sequencing facilities (Macrogen, Seoul, Republic of Korea). Clone Manager 10 (Sci-Ed Software, Colorado, USA) (10) was used to pair and merge the forward and reverse Sanger reads. The target genome was sequenced with fourfold coverage using the Sanger technique. The NCBI GenBank database (http://www.ncbi.nlm.nih.gov/) was used to obtain the nucleotide sequences for all eight segments to construct a phylogenetic tree containing the eight viral genes (11). The genes were aligned using ClustalW, implemented in MEGA 11 (12). A phylogenetic tree was generated using the neighbor-joining approach with 1,000 bootstrap replicates. Although there were 1,000 bootstrap replicates for each node, only percentage values greater than 60% were accepted. All tools were run with default parameters, unless otherwise specified.

**Fig 1:**
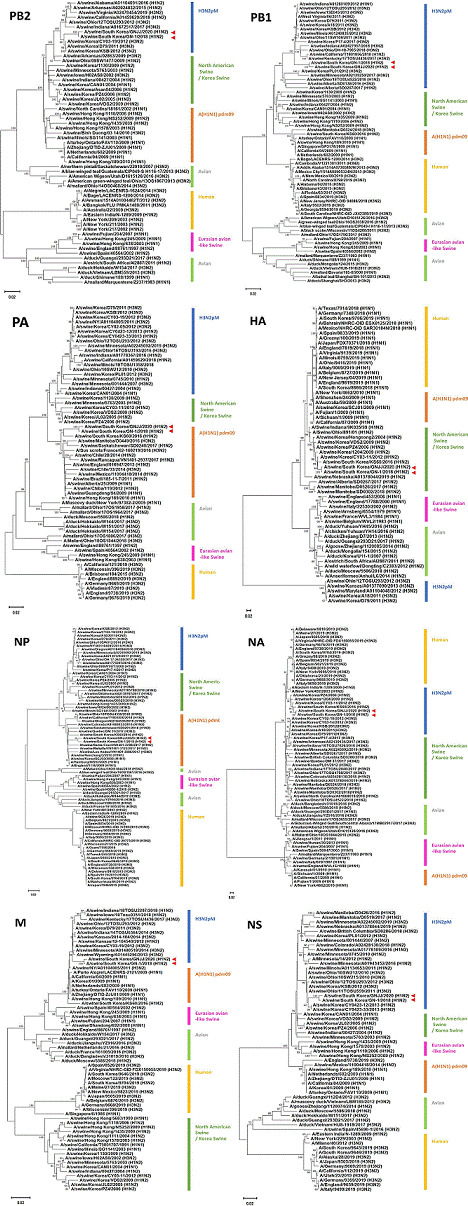
Phylogenetic analysis based on the eight gene sequences of the influenza A viruses, “A/Swine/South Korea/GN-1/2018” and “A/Swine/South Korea/GNJJ/2020.” Trees were generated using MEGA 11 for 1,000 bootstrap replicates, yielding only values greater than 60%. The two viruses are indicated by red triangles in each panel. The scale bars correspond to a phylogenetic distance of 0.02 nucleotide substitutions per site.

Upon aligning the eight genes of the two viruses sequenced in this study, a notable 99% similarity was observed. The HA and NA sequences were remarkably similar to those of the H1N2 strains, “A/swine/Korea/CY03-11/2012 (H1N2)” and “A/swine/South Korea/s802/2018 (H1N2),” respectively, that were isolated from Korean pigs (Table 1). According to the phylogenetic analysis, PB2, PB1, NA, M, and NS belong to the H3N2pM lineage, whereas PA and NP belong to the A (H1N1) 2009 pandemic lineage. The HA genes of the two viruses were assigned to the North American swine virus lineage (Fig. 1).

**TABLE 1:** Influenza A viruses with the highest nucleotide homogeneity with A/Swine/South Korea/GN-1/2018 (H1N2) and A/Swine/South Korea/GNJJ/2020 (H1N2)

Gene	Viruses	GC content (%)	Size (bp)	Sequence similarity (%)	Closest related viruses		
					Viruses	Accession no.	Identity (%)
PB2	GN-1/2018	44.7	2,280	99.3	A/swine/Korea/CY03-19/2012 (H3N2)	KC471486.1	97.9%
	GNJJ/2020	44.9					97.2%
PB1	GN-1/2018	42.1	2,374	99.3	A/West Virginia/06/2011 (H3N2)	JQ290162.1	97.6%
	GNJJ/2020	41.7					97.1%
PA	GN-1/2018	43.5	2,151	99.3	A/swine/South Korea/s802/2018 (H1N2)	MN094314.1	97.1%
	GNJJ/2020	43.5			A/swine/Korea/SCJ09/2009 (H1N1)	HM189618.1	96.3
HA	GN-1/2018	40.2	1,701	98.9	A/swine/Korea/CY03-11/2012 (H1N2)	KC471425.1	96.7
	GNJJ/2020	40.5					96.0
NP	GN-1/2018	45.5	1,515	98.6	A/swine/Nanchang/3/2010 (H1N1)	JF275920.1	97.2
	GNJJ/2020	45.6			A/Hamburg/INS92/2009 (H1N1)	CY057353.1	97.0
NA	GN-1/2018	41.6	1,410	99.2	A/swine/South Korea/s802/2018 (H1N2)	MN094317.1	96.6
	GNJJ/2020	6					98.2
M	GN-1/2018	46.7	982	99.6	A/swine/Korea/CY03-19/2012 (H3N2)	KC471492.1	98.6
	GNJJ/2020	46.7					98.4
NS	GN-1/2018	44.5	838	99.2	A/swine/North Carolina/A01291004/2013 (H1N1)	KY056425.1	98.4
	GNJJ/2020	44.4					98.1

## Data Availability

For A/swine/South Korea/GN-1/2018 (H1N2), the GenBank accession numbers are OP804182–OP804189 and the SRA accession number is SRR23634035. For A/swine/South Korea/GNJJ/2020 (H1N2), the GenBank accession numbers are OP804222–OP804229 and the SRA accession number is SRR23634029. Both viruses were deposited under BioProject number PRJNA938163 and BioSample number SAMN33427201.
